# Editorial: Visual perception in children and adolescents with visual impairments

**DOI:** 10.3389/fnhum.2025.1713145

**Published:** 2025-10-29

**Authors:** Corinna M. Bauer, Jeff C. Rabin, Karen Harpster

**Affiliations:** ^1^Massachusetts General Hospital, Boston, MA, United States; ^2^Harvard Medical School, Boston, MA, United States; ^3^Rosenberg School of Optometry, University of the Incarnate Word, San Antonio, TX, United States; ^4^Cincinnati Children's Hospital Medical Center, Cincinnati, OH, United States; ^5^University of Cincinnati, Cincinnati, OH, United States

**Keywords:** visual impairment, cerebral/cortical visual impairment (CVI), quality of life, visual perception, vision assessment, persons with lived experience

## Introduction

The use of functional vision to perceive and interact with one's surroundings and the outside world can be compromised in individuals with visual impairments. Thus, visual impairment—whether due to ocular causes, cerebral visual impairment (CVI), or both—may negatively affect one's activities and participation within the domains of the International Classification of Function (Deramore Denver et al., 2016). Therefore, receiving appropriate services, interventions, and/or supports to gain veridical access to the environment is essential for those with visual impairments. The articles in this Research Topic focus on these unique challenges and highlight how appropriate accommodations and interventions may improve meaningful access and quality-of-life.

As outlined by McDowell et al., there remain a number of challenges regarding our understanding of the visual and perceptual dysfunctions and challenges that are experienced by those with CVI. Consequently, they often go unrecognized, resulting in children and/or adults with CVI-related behaviors who may receive inappropriate diagnostic labels and ineffective educational, vocational habilitation, and/or therapeutic interventions that might not be tailored to one's unique needs. This review underscores the necessity of fully characterizing and diagnosing the visual capacities and challenges of the individual, coupled with assistance, accommodations and interventions needed for an individual with CVI to obtain meaningful access to educational or vocational materials.

## Characterizing at-risk populations

CVI (i.e., visual impairment and/or dysfunction secondary to brain injury, damage, malformation, or malfunctioning of the brain's visual processing centers) is increasingly being recognized as a common sequalae across various clinical populations, including individuals with genetic disorders and conditions. In their article, Boatwright et al. presents data from a case series of three individuals with Down Syndrome (DS) and CVI with specific challenges in visual function as well as functional vision of the ventral stream, dorsal stream, and attention accompanied by subcortical changes and abnormal EEGs. While ocular conditions are well-documented in the DS population, CVI is only recently being considered for this population. As such, the CVI diagnosis may come later in life, as was the case for the three cases presented by Boatwright et al.. The authors note that receiving the CVI diagnosis for these families validated their concerns regarding their children's vision use. Earlier detection in such patients can indeed improve cognitive functioning required to optimally develop and interact with the world around them.

## Supporting children with brain-based visual impairments

Jakubowski et al. invited family members and specialists who work with children with brain-based visual impairments to participate in an online survey. The survey was completed by educators, rehabilitation staff, clinicians, and family members. Results showed that although caregivers felt like undervalued members of the team, Professionals designated them as the principal and most credible informants regarding information about their child's visual status. There was also consensus that transdisciplinary clinics and inter-professional care were impactful and provided client centered care. Finally, respondents felt that CVI was underdiagnosed, and more formal training was needed to advance the field and provide adequate support to those with brain-based vision impairment.

## Methods for evaluating functional vision

Currently, a large body of research involving CVI focuses on the use of surveys and structured history taking, such as the Flemish CVI Questionnaire (Ortibus et al., 2011), the CVI (or Insight) Inventory (Ghahghaei et al., 2021; Fazzi et al., 2007), and the Teach CVI Screening Tool (Teach CVI Partnership, 2017). There is tremendous value in these approaches as they can provide invaluable information regarding the daily challenges in the functional use of vision (Dutton et al., 2010). They can also provide insight into the aspects of visual functioning that are not affected for an individual with CVI. Research reported by Martin et al. applied Rasch analysis to the Teach-CVI Screening Tool in order to evaluate the relationship between person and item measures from the Likert responses. Their results suggest that the tool is well targeted (i.e., relative absence of floor and ceiling effects) and reflects a single underlying construct, namely “the impact of CVI on visual ability” (Martin et al.).

In addition to research involving questionnaires and surveys, there are also concerted efforts by many in the field of vision research, and CVI in particular, to more rigorously characterize visual ability in those with or at risk for CVI (Mooney et al., 2021, 2018, 2020; Kooiker et al., 2016; Hokken et al., 2024, 2025; Bennett et al., 2018a,b; Pamir et al., 2024; Ben Itzhak et al., 2021, 2025; Bauer et al., 2023; Walter et al., 2025). Most recently, approaches implement eye tracking-based methods, which do not rely on a verbal or manual response and, as such, may be more widely applicable to the spectrum of abilities in individuals with CVI. Among these is the manuscript in this Research Topic by Matsunaga et al., which outlines their proposed method for using AI-enabled image saliency analysis to evaluate whether children with or suspected of CVI gaze at similar image features.

Two aspects of CVI frequently studied are selective visual attention impairments and dorsal stream dysfunction. In the article by Hokken et al., they specifically tested global visual selective attention via Gestalt, Navon, and Kanizsa figures in school-aged children. These tasks require the ability to integrate visual elements in order to perceive a whole visual representation. Children with CVI demonstrated global impairments on all tasks compared to neurotypical children as well as children with ADHD or dyslexia. While the authors suggest their findings support the dorsal stream dysfunction hypothesis, the data also reflect underlying impairments in mid-level visual perceptual dysfunctions, such as contour integration and figure-ground segmentation. However, it has yet to be determined if mid-level and dorsal stream dysfunctions can be disentangled in CVI, particularly as dorsal stream dysfunction may manifest as simultanagnostic vision (inability to see more than a single object at one time) and/or restriction of visual attention to small portions of the visual scene. These difficulties are highlighted by three cases in the article by St Clair Tracy et al.. Based on the reported experiences, the authors generated a series of simulations for simultanagnostic vision, demonstrating how the functional field of view may be impacted by the presence of visual clutter. The cases also illustrate the potential emotional responses to complex environments as well as the potential benefit of mindfulness exercises. Additional research is required to fully appreciate how conscious vision strategies and mindfulness exercises impact the ability to expand the dynamic functional field of view and patient wellbeing.

## Impact of visual impairments on quality of life and wellbeing

Visual impairment may have an impact on the level of satisfaction and participation in daily activities of teenagers. In the qualitative study of 25 teenagers with either visual or motor impairments, Veldhorst et al. conducted semi-structured interviews which revealed 13 themes, many of which were shared between those with visual and motor impairments. Notably, visual impairment here was based on visual acuity and/or visual field restriction and did not specifically extend to teenagers with cerebral visual impairment (CVI), and the cause of motor impairment is not specified. However, motor impairments were classified based on the GMFCS. Overall, the teenagers in both groups reported satisfaction with the level and amount of participation in leisure activities, with notable differences in barriers to participation. Increasingly visual dysfunctions are being reported and recognized in individuals with cerebral palsy and developmental coordination disorder, with visual impairment occurring over three times more frequently than in the general population (Lo Cascio, 1977; Fazzi et al., 2007, 2012; Duke et al., 2020; Chokron and Dutton, 2016). However, participants with co-occurring visual and motor impairments were excluded from this study. Thus, it remains unclear how barriers and facilitators related to activities and participation are affected in this population.

## Improving outcomes—evidence from persons with lived experience

Understanding CVI based on the lived experience of individuals with CVI and their caregivers can be extremely impactful and provides invaluable insight. Bennett et al. emphasized that visual perception experiences and the development of concepts are different for each individual with CVI. They described how individuals with CVI use compensatory skills and strategies to perceive objects, access their environments, and socialize. Duesing et al. describe three case studies of individuals with early onset CVI, and interventions used to improve visual perception abilities and the understanding of the world around them. The authors describe experiences and strategies using various approaches, including self-taught compensatory strategies, sensory substitution, and augmentation techniques, to improve meaningful access to their environment. Importantly, because CVI manifests differently in each person with CVI, intervention approaches need to also be uniquely tailored to each patient and should be regularly evaluated for efficacy (Lueck et al., 2019, 2023).

## Future directions—concluding thoughts

Although CVI research is advancing, the current body of evidence remains limited. There are many novel ideas and strategies still in their emerging phasea. In particular, studies related to early detection and early intervention are needed, facilitated by greater inter-disciplinary careRigorous intervention studies employing control groups are essential for advancing scientific knowledge and informing best practices in clinical care. Additionally, to strengthen the field, there is a pressing need for the development and validation of comprehensive outcome measures, both objective and subjective, across the spectrum of abilities and challenges, particularly those that assess not only clinical gains but also functional outcomes in daily activities and participation. As outlined by Jakubowski et al.—we still do not know enough.
